# Undifferentiated pleomorphic sarcoma of the pancreas diagnosed by EUS–guided fine-needle biopsy

**DOI:** 10.1097/eus.0000000000000184

**Published:** 2026-05-26

**Authors:** Liliangzi Guo, Qi Tang, Qiufeng Lin, Defeng Li, Lisheng Wang, Jun Yao

**Affiliations:** Department of Gastroenterology, Shenzhen People’s Hospital (The First Affiliated Hospital, Southern University of Science and Technology; The Second Clinical Medical College, Jinan University), Shenzhen, Guangdong Province, China.

A 72-year-old patient was admitted to our hospital due to facial edema for 1 month. Laboratory test found that white blood cell was 8.92 × 10^9^/L, hemoglobin 98 g/L, lymphocyte% 11.8%, creatinine 181 µmol/L, lomerular filtration rate 31.7 mL/min/1.73 m^2^, and liver function and tumor markers were normal. Positron emission tomography/computed tomography revealed a mass with increased glucose metabolism in the body of the pancreas, with cancer thrombus in the superior vena cava and multiple lymph node metastases, which was considered to be malignant [Figure 1]. EUS showed a hypoechoic, well-demarcated, about 4.4-cm diameter mass in the body of the pancreas, with dilation of the pancreatic duct and common bile duct [Figure 2A–C]. Endoscopic ultrasound-guided fine-needle biopsy (EUS-FNB) was performed with a 22-G needle, with a total of 3 passes [Figure 2D]. On immunostaining, the tumor was positive for Vimentin, Trypsin, SMARCA4, and CDK4, negative for CK, HMB45, CD34, S100, and Desmin. The tumor Ki-67 expression was about 30% [Figure 3]. Based on the morphology and immunohistochemical staining, the tumor was diagnosed as an undifferentiated pleomorphic sarcoma (UPS) of the pancreas. The patient refused further treatment and was discharged 6 days later.

**Figure 1.**
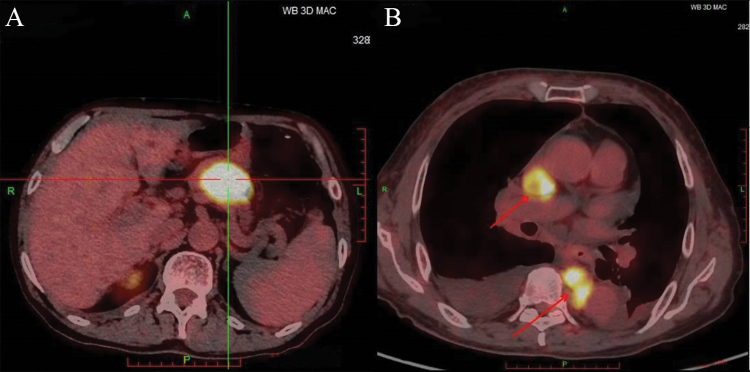
A, PET/CT revealed a mass with increased glucose metabolism in the body of the pancreas. B, Cancer thrombus in the superior vena cava, which caused the facial edema. PET/CT, positron emission tomography/computed tomography.

**Figure 2.**
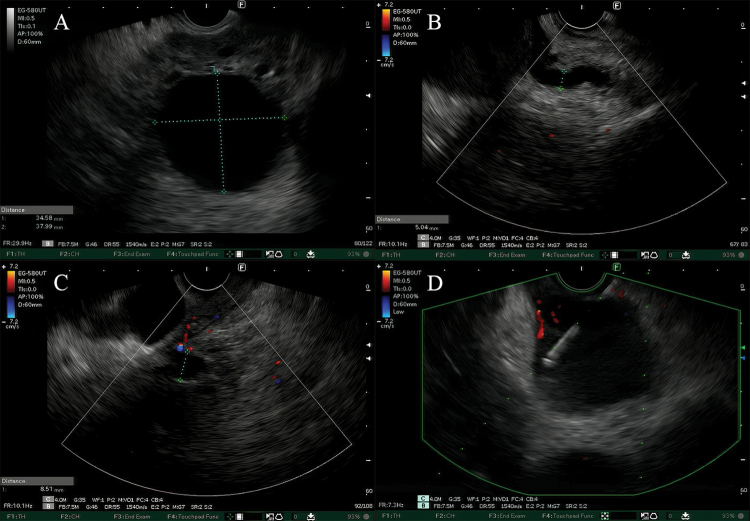
A, EUS showed a hypoechoic, well-demarcated, about 4.4-cm diameter mass in the body of the pancreas. B, Dilation of the pancreatic duct. C, Dilation of the common bile duct. D, EUS-FNB was performed with a 22-G needle. EUS-FNB, EUS-guided fine-needle biopsy.

**Figure 3.**
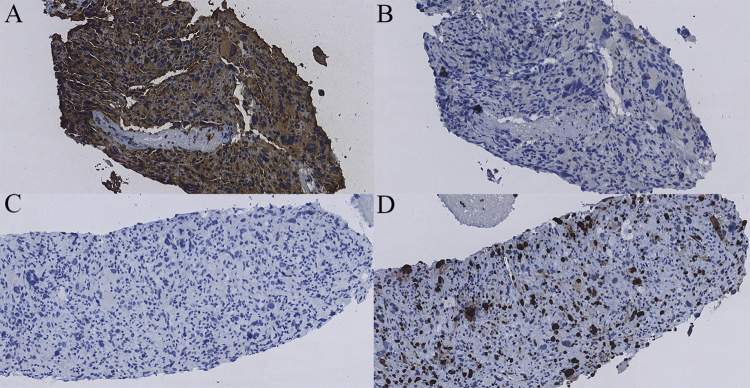
Immunohistochemical findings. A, Vimentin (+). B, CK (−). C, Desmin (−). D, Ki-67 index was about 30%.

UPS is a malignant fibrous histiocytoma that often occurs in limbs, trunk, and retroperitoneal tissues. However, the incidence of UPS involving the digestive tract is extremely rare, and in the pancreas is even lower, accounting for only 0.1%.^[1]^ Pancreatic UPS is an extremely rare and highly malignant tumor, which is difficult to diagnose without obtaining tumor tissues. This is the first report of pancreatic UPS diagnosed by EUS-FNB. Radical resection is the mainstay of treatment in primary localized retroperitoneal sarcomas.^[2]^ However, for patients with pancreatic mass that are difficult to identify and preclude surgical intervention, EUS-FNB is worthy to recommend because it can get enough tumor tissues for immunohistochemistry.

## Source of Funding

The study was supported by the National Natural Science Foundation of China (82400704) and the Technical Research and Development Project of Shenzhen (JCYJ20240813104413019).

## Ethical Statements

This study was approved by the Ethics Committee of Shenzhen People’s Hospital. The authors certify that they have obtained all appropriate patient consent. The patient has given his consent for his images and other clinical information to be reported in the journal.

## Conflicts of Interest

The authors declare that they have no conflict of interest with regard to the content of this report.

## Author Contributions

J. Yao and L. Wang contributed to the study conception and design. Material preparation, data collection, and analysis were performed by L. Guo, Q. Tang, and Q. Lin. The draft of the manuscript was written by L. Guo. The manuscript was reviewed and revised by D. Li. All authors read and approved the final manuscript.

## Data Availability Statement

Data sharing not applicable to this article as no datasets were generated or analyzed during the current study.
